# Native multi-qubit gates in transmon qubits via synchronous driving

**DOI:** 10.1038/s41598-024-76396-9

**Published:** 2024-10-29

**Authors:** Sagar Silva Pratapsi, Diogo Cruz, Paulo André

**Affiliations:** 1 Instituto Superior Técnico, Universidade de Lisboa, Lisbon, Portugal; 2 Instituto de Telecomunicações, Lisbon, Portugal

**Keywords:** Qubits, Superconducting devices

## Abstract

Quantum computation holds the promise of solving computational problems which are believed to be classically intractable. However, in practice, quantum devices are still limited by their relatively short coherence times and imperfect circuit-hardware mapping. In this work, we present the parallelization of pre-calibrated pulses at the hardware level as an easy-to-implement strategy to optimize quantum gates. Focusing on $R_{ZX}$ gates, we demonstrate that such parallelization leads to improved fidelity and gate time reduction, when compared to serial concatenation. As measured by Cycle Benchmarking and Process Tomography, we reduce gate errors by half. We show that this strategy can be applied to other gates like the CNOT and CZ, and it may benefit tasks such as Hamiltonian simulation problems, amplitude amplification, and error-correction codes.

## Introduction

The field of quantum computing has seen an explosion of interest in the recent decades ^1–3^. Seminal results such as Shor’s algorithm ^3–5^ and the threshold theorem for Quantum Error Correction ^6–9^ have provided the hope that quantum computers may one day solve problems that are currently intractable^3^.

One major limitation to the field of quantum computation is that the quantum circuit abstraction breaks down when one uses real quantum devices. Physically, unitaries are only implemented approximately, using classical fields, and the resulting operation will not match the expected one with perfect fidelity^10,11^. When designing quantum circuits, the circuit implementation is generally not tailored to some particular hardware (with the notable exception of some hardware-specific variational algorithms^12–15^). The circuit gates may be mapped sub-optimally to the pulse-level instruction schedule, thereby leading to longer pulse instructions and higher decoherence^16–20^.

In fact, another main limitation of noisy intermediate-scale quantum (NISQ) devices is their relatively short coherence times ^21^. As the physical apparatus encoding the quantum state quickly interacts with the environment and suffers decoherence, there is a limited window where quantum operations can be performed on the quantum state, while still retaining acceptable fidelity. The rapid decoherence imposes limits on the circuit depths that are practically viable, and the number of gates applied, which limits the complexity of the quantum circuits that can be implemented in practice. This constraint ultimately limits the practical usefulness of quantum computing to solve problems where there is an expected theoretical quantum advantage. Consequently, in order to make the most use of current NISQ devices, we are compelled to shorten circuit times whenever possible, thereby enabling us to implement more complex circuits in the same time window.

Various approaches have been proposed to map abstract quantum circuits to efficient and fast pulse-level instructions. Transpilation, for instance, relies on gate cancellation and simplification, using optimization techniques^22^, and takes into account known connectivity constraints in the quantum device of interest, leading to a mapping that requires fewer intermediate gates to provide the desired connectivity, such as SWAP gates. At a lower level, circuit compilation^23,24^ tries to decompose the original quantum circuit in terms of quantum gates that are less common but more representative of the underlying physical system. Finally, at the lowest level, we may use quantum control^25^ and work with the pulse-level implementation directly, which might be the optimal policy to ensure high fidelities. However, this comes with significant trade-offs. The pulses that can be directly implemented in the superconducting qubits, for instance, do not map to commonly used quantum gates^11^. As a result, building large quantum circuits by using these pulse gates directly would be unintuitive and impractical.

In this work, we present an optimization strategy that lies between compilation and quantum control and that is readily available in contemporary devices—parallelization of pre-calibrated pulses. Following this procedure, we are able to map unitaries to native instructions, reduce gate times and increase fidelity, all with minimal effort. Recent independent work^26^ followed a similar approach to implement multi-qubit parity gates. While both studies leverage simultaneous cross-resonance drives, there are some key differences in focus and methodology. Our work provides a more generalized framework for parallelizing $R_{ZX}$ gates, which we extend theoretically to *n*-qubit systems. In contrast,^26^ focus specifically on three-qubit parity gates and provide a detailed Hamiltonian analysis of the simultaneous cross-resonance drives. Both studies demonstrate improved gate fidelities, with^26^ using interleaved randomized benchmarking and our work employing both process tomography and cycle benchmarking. While^26^ concentrate on applications in quantum error correction, particularly for the heavy-hexagon code, our work explores additional applications in Hamiltonian simulation and amplitude amplification.

We show in section 2 that many Hamiltonian simulation problems can benefit from such a parallelization. One particular realization of this strategy relies on parallelizing $R_{ZX}(\theta )$ gates with a shared qubit. In section 3 we present the parallelization strategy and demonstrate that the Parallel $R_{ZX}$ gate, $P_{abc}(\theta )$, is natively available in real devices and that it outperforms its serial counterpart. In fact, as measured by Cycle Benchmarking, by parallelizing two $R_{ZX}(\pi /2)$ we obtain a fidelity of $99.15(3)\%$, corresponding to half the error of its serial counterpart. We emphasize that the $P_{abc}(\theta )$ gates constitute a family of native high-fidelity three-qubit gates. Finally, in section 4, we discuss how the parallelization strategy can be further generalized to CNOT and CZ gates, and applied to other problems.

## Pulse parallelization in NISQ Devices

**Fig. 1:**
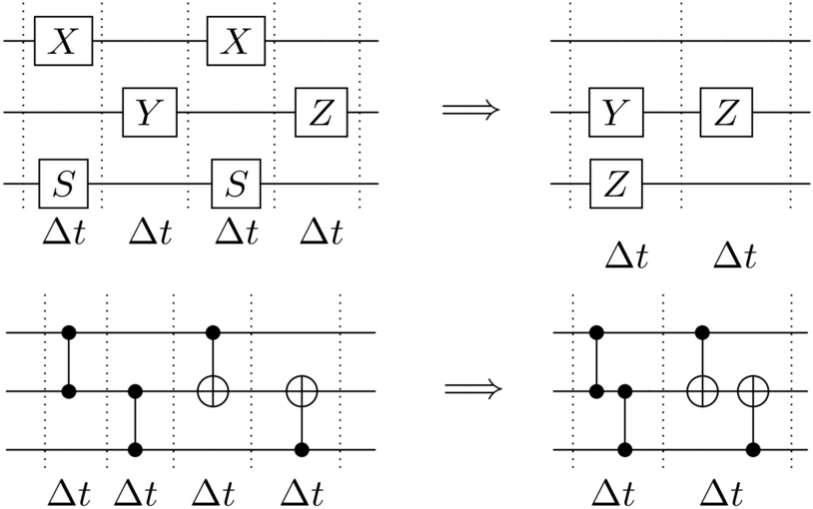
**(Top)** Standard optimization techniques already simplify single qubit gate implementations, by canceling gates (as in the top qubit, where $X^2$ is simplified to the identity), merging them ($S^2 \rightarrow Z$), or simply parallelizing them at the pulse level (the pulse-level operations in the middle and bottom qubits go from being implemented in series to being in parallel). **(Bottom)** While canceling and merging are also common techniques to simplify multi-qubit gates, parallelization is generally only performed if the gates share no qubits. In this work, we show that, by parallelizing the low-level pulses that define the complex multi-qubit gates, some overlapping gates can be implemented in parallel. As multi-qubit gates are the main contributors to the circuit duration, considerable time savings are possible, without significant overheads.

Current quantum devices already heavily rely on parallelization of single qubit gates in order to achieve short circuit depths and durations (see fig. 1). In this case, the pulse-level parallelization is straightforward, as there is no pulse overlap between qubits. Similarly, multi-qubit gates can be parallelized at the pulse level if no qubits are shared among them. However, in order to harness the full advantage of quantum devices, highly entangled quantum states are required, and to create these it is necessary to apply multi-qubit gates to shared qubits, for which parallelization at the pulse level is no longer straightforward.

Fortunately, while multi-qubit gates applied to overlapping qubits cannot be parallelized at the abstract circuit layer, we show that it is possible to parallelize them at the pulse layer, and that this parallelization is straightforward to implement once recognized.

Two gates $U_1, U_2$ may be parallelizable, for instance, if they are generated by two commuting time-independent Hamiltonians $H_1$ and $H_2$,

1 $$\begin{aligned} U_1 U_2 = e^{-iH_1 t_1} e^{-iH_2 t_2} = e^{-it(H_1' + H_2')}. \end{aligned}$$

Here, $H_i' = H_i t_i / t$ are re-scaled versions of the Hamiltonians. The evolution on the right-hand side happens in time *t*, which may be chosen to be less than $t_1 + t_2,$ as illustrated in fig. 1.

In actual quantum machines—using superconducting transmon qubits, trapped ions, or others—Hamiltonians are implemented via an external driving signal, like an electrical current or a laser^11^. Therefore, if two gates commute, it is possible that their underlying pulse signals can be superposed in order to approximate the evolution in eq. (1). Of course, it is important to emphasize that in real devices we can only approximate some desired Hamiltonian, as there are usually extra interactions that break commutativity^16–20^. Additionally, implementing $H_1' + H_2'$ may consume more resources than $H_1$ and $H_2$ individually, and there will be practical limitations to its implementation, like voltage saturation or non-linear effects. Nevertheless, if *t* is less than $t_1 + t_2$, parallelization may help achieve a computation in a shorter time interval, relative to the system’s decoherence time, resulting in a net fidelity gain.

For this reason, it is interesting to ask whether pulse superposition can be used for gate parallelization in practice. We show that, in contemporary commercially-available quantum devices, the answer is positive.

### Hamiltonian simulation with $R_{ZX}$ gates

To motivate the use of parallel gates with an application, consider a general *n*-qubit Hamiltonian composed of Pauli terms of the form $h_i O_{i_1} \cdots O_{i_k}$ with $O \in \{X,Y,Z\}$ and $\{i_1,\ldots ,i_k\}$ ($k\le n$) an ordered subset of $\{1,\ldots , n\}$, and $h_i$ real constants. Since $\{I, X, Y, Z\}^{\otimes n}$ forms an operator basis, any Hamiltonian may in fact be decomposed in this way. Using trotterization techniques^27^, we may implement the Hamiltonian evolution by evolving Pauli terms separately.

For now, we analyze the simple case where two Pauli terms commute, which will allow us to parallelize their evolution following eq. (1). Consider then, as an example, the three-qubit Hamiltonian acting on qubits *a*, *b*, *c*, 

2 $$\begin{aligned} H&= h_1 O_a O_b + h_2 O_b O_c,\end{aligned}$$

3 $$\begin{aligned} \text {with } O&\in \{X,Y,Z\}. \end{aligned}$$

A simple change of basis at qubits *a*, *b*,  and *c* may convert *H* into

4 $$\begin{aligned} \tilde{H}&= U H U^\dagger \end{aligned}$$

5 $$\begin{aligned}&= h_1 Z_a X_b + h_2 X_b Z_c \end{aligned}$$

where *U* is the tensor product of single-qubit Pauli basis-change operators, given by the relation $\sqrt{X} Y \sqrt{X}^\dagger = Z$ and its cyclical permutations under (*X*, *Y*, *Z*).

An $R_{ZX}$ gate, acting on a control qubit *a* and a target qubit *b*, is the unitary generated by $Z_a X_b$,

6 $$\begin{aligned} R_{Z_a X_b}(\theta ) = \exp (-i (\theta /2) Z_a X_b). \end{aligned}$$

By using $\tilde{H}$ and *U*, the evolution of *H* in eq. (2) can be implemented with the gate $P_{abc}(h_1t, h_2t)$, given by

7 $$\begin{aligned} P_{abc}(\theta _1, \theta _2)&: = \exp (-i\ [ \frac{\theta _1}{2}Z_aX_b + \frac{\theta _2}{2}X_bZ_c ]) \end{aligned}$$

8 $$\begin{aligned}&= R_{Z_a X_b}(\theta _1) \, R_{Z_c X_b}(\theta _2). \end{aligned}$$

For the sake of simplicity, we also define $P_{abc}(\theta ) := P_{abc}(\theta , \theta )$. With this concise notation, the *a* and *c* indices always refer to the *Z* gate, and the *b* index to the *X* gate.

The $R_{ZX}$ gate is then a candidate for parallelization.

As an application, consider the Heisenberg Hamiltonian

9 $$\begin{aligned} H = X_1 X_2 + X_2 X_3 + Y_1 Y_2 + Y_2 Y_3 + Z_1 Z_2 + Z_2 Z_3. \end{aligned}$$

Applying the following Trotterization to its evolution

10 $$\begin{aligned} (e^{-i\frac{t}{n}(X_1X_2+X_2X_3)} e^{-i\frac{t}{n}(Y_1Y_2+Y_2Y_3)} e^{-i\frac{t}{n}(Z_1Z_2+Z_2Z_3)})^n \end{aligned}$$

reveals a case where the parallelization technique can be applied. Each term can be converted, using the single qubit gates from eq. (4), to the unitary

11 $$\begin{aligned} e^{-i\frac{t}{n}(Z_1X_2+X_2Z_3)}, \end{aligned}$$

which can be seen as a parallelized version of two $R_{ZX}(t/n)$ gates.

In the following section, we demonstrate that parallelizing two $R_{ZX}$ gates indeed leads to a fidelity gain in current quantum devices. In fact, the $R_{ZX}$ gate is quite versatile and can be used to construct other gates, such as the $\textrm{CNOT}$ gate, which can also be parallelized, as we will explore in section 4.

Although we demonstrate our approach for the three-qubit case, it can be straightforwardly generalized to $(n+1)$ qubits. Considering one qubit to be the target *t*, and the other *n* qubits the controls $c_i$, any Hamiltonian of the form

12 $$\begin{aligned} H = \sum _{i=1}^n h_i O_{c_i} O_t \end{aligned}$$

can be directly implemented by a pulse-parallelized version of the gate

13 $$\begin{aligned} P_{t,{\textbf {c}}}(\varvec{\theta })&= \exp (-i\sum _{i=1}^n\frac{\theta _i}{2}Z_{c_i}X_t),\end{aligned}$$

14 $$\begin{aligned} \text {with }\varvec{\theta }&= (\theta _1,\ldots ,\theta _n) = (h_1t,\ldots ,h_nt)\end{aligned}$$

15 $$\begin{aligned} \text {and } {\textbf {c}}&= (c_1,\ldots , c_{n}). \end{aligned}$$

Similarly, we define $P_{t,{\textbf {c}}}(\theta ) := P_{t,{\textbf {c}}}(\theta ,\ldots ,\theta )$.

So far, we have only considered the case where the target is the shared qubit. For generalized versions, see Appendix C. With this generalized notation, the control and target indices always refer to the indices of the *Z* and *X* gates, respectively.

We can now demonstrate that this parallelization strategy is feasible in contemporary quantum devices.

## Demonstration of a parallel $R_{ZX}$ gate

In this Section, we demonstrate the implementation of $P_{abc}$ (see eq. (7)) in a platform of superconducting qubits, IBM’s ibmq_belem device.

We emphasize that the implementation we describe below relies on pre-calibrated pulses provided by the experimental platform. It thus requires minimal effort to set and is accessible on commercially available quantum devices.

### Transmon Hamiltonians

The two-transmon system driven by the CR pulse at qubits *a* and *b* is approximately described^18^ by the time-independent Hamiltonian

16 $$\begin{aligned} H_\textrm{CR}&= \frac{Z_aB_b + I_a C_b}{2}\end{aligned}$$

17 $$\begin{aligned} B_b&= \omega _{ZI} I_b + \omega _{ZX} X_b + \omega _{ZY} Y_b + \omega _{ZZ} Z_b\end{aligned}$$

18 $$\begin{aligned} C_b&= \omega _{IX} X_b + \omega _{IY} Y_b + \omega _{IZ} Z_b, \end{aligned}$$

where *I* is the identity matrix and *X*, *Y*, *Z* are the Pauli matrices. The symbols $\omega _{(\cdot )}$ are real coupling constants. We wish to isolate the main $Z_aX_b$ term. By carefully picking the phase of the CR pulse and applying a compensation pulse to the target qubit, undesirable terms in the Hamiltonian can be suppressed. Additionally, echo sequences can be employed to mitigate coherent errors (see Appendix B2).

A more detailed description of these noise suppression techniques can be found in^16^, and brief introductions in^17–20^. By suppressing these terms, we end up with the Hamiltonian

19 $$\begin{aligned} H_{ZX} = \frac{Z_aX_b}{2}, \end{aligned}$$

allowing us to implement the $R_{ZX}[a][b](\theta )$ gate, given by eq. (6).

Obtaining the pulse composition of $R_{ZX}[a][b](\theta )$ for a general $\theta$ can be done from the calibrated pulses used for the CNOT gate, as explained in the next Section.

**Fig. 2:**
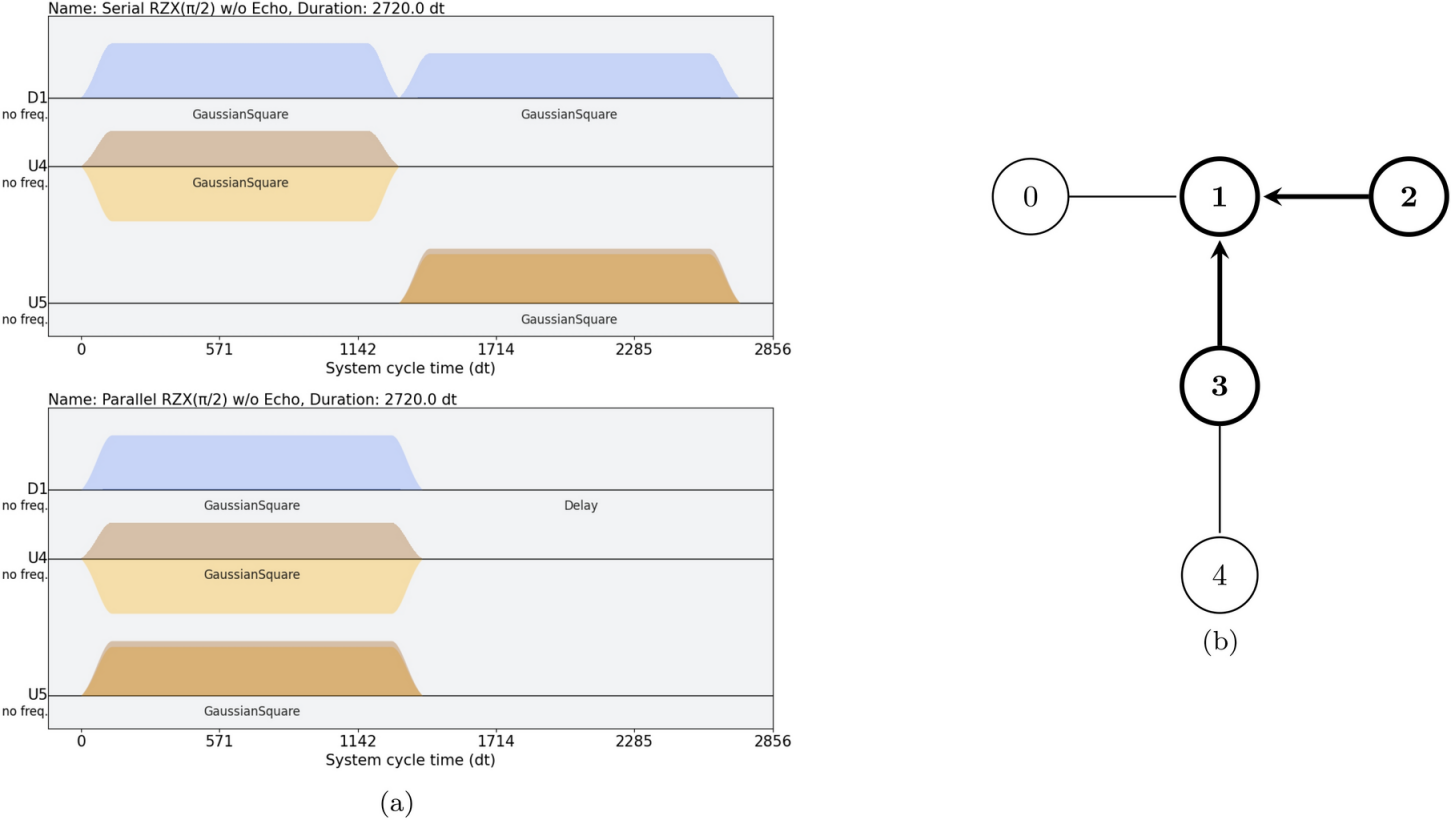
**(a)** Example pulse sequence for the serial **(top)** and parallel **(bottom)** version of the double $R_{ZX}$ gate, as described in section 3.2, for the ibmq_belem device. By parallelizing the CR pulses (orange) and merging the compensation tones (blue), the gate duration of two $R_{ZX}$ gates may be reduced by up to half. The parallelization can be made automatically, without additional calibration schemes, by using the $R_{ZX}$ gate pulse shape stemming from the associated CNOT calibration, as described in section 3.2; **(b)** Layout of the intervening qubits for the two $R_{ZX}$ gates, in ibmq_belem. The nodes, and their labels, represent the device’s qubits, while the edges indicate the possible direct two-gate implementations. In bold, we highlight the intervening qubits for the pulses on the left; the arrows represent the $R_{ZX}$ gates, with the arrowhead pointing towards the target qubit. These gates are implemented with two-qubit CR pulses (U4 and U5 channels, corresponding to the connections (2,1) and (3,1), with qubit 1 acting as target in both cases) and compensation tones (D1 channel).

### Parallel $R_{ZX}$ pulse calibration and design

#### Pulse calibration from existing $\textrm{CNOT}$ gates

As the CNOT gate (with echo, see Appendix B2) is widely used as a standard for 2-qubit entangling quantum gates, the calibration of current quantum devices generally attempts to mitigate the error present in CNOT gate applications. Since the echoed CNOT gates are implemented through the use of cross-resonance (CR) pulses with angle $\theta = \pm \pi /4$, pulses of this format are expected to present lower error rates, and can be used to define suitable pulses for other angle values.

Often, the echoed CNOT gate implementation is achieved by first decomposing the gates in terms of $R_{ZX}(\pi /4)$ (or $R_{ZX}(\pi /2)$ without echo) gates and additional single qubit gates. The error of the CNOT gates is then minimized by calibrating the pulses of the constituting gates, according to the decomposition used. There are several equivalent ways of decomposing CNOT gates in this manner. A possible decomposition for the unechoed CNOT gate is20which is the one often used on IBM’s quantum devices. An alternative formulation is21The echoed variant (which may be directly used in the calibration) can be defined by using the echoed version of the $R_{ZX}(\pi /2)$ gate, as explained in Appendix B2.

Finding calibrated pulses for the $\textrm{CNOT}$ gate indirectly determines calibrated pulses for $R_{ZX}(\pi /2)$, from which the pulses for $R_{ZX}(\theta )$ (for $\theta$ other than $\pi /2$) can be set.

For $R_{ZX}(\pi /2)$, in addition to the main cross-resonance pulse tones, there are also compensation tones applied to the target qubit during the main pulse. On IBM’s devices, both types of pulses have the shape of a “Gaussian square”, that is, a pulse that initially ramps up at $t_i$ following a squared exponential function until reaching a peak amplitude $A_\textrm{CR}$, then plateaus at that amplitude for a set period of time, before ramping down to zero at $t_f$ using a similar squared exponential. See fig. 2a , 2b for examples, and^28,29^ for a more thorough description. Let $t_\textrm{CR} = |t_f - t_i|$ be the total pulse duration. The angle $\theta$ implemented by these pulses is proportional to the integral of the pulse. That is, if the pulse amplitude at time *t* is *A*(*t*), then

22 $$\begin{aligned} \theta \propto \int _{t_i}^{t_f} A(t) \,\textrm{d}t. \end{aligned}$$

If the ramping up and down portions are negligible, then this relation may be simplified to

23 $$\begin{aligned} \theta \propto A_\textrm{CR} t_\textrm{CR}. \end{aligned}$$

Therefore, by modifying either the duration $t_\textrm{CR}$ or the peak amplitude $A_\textrm{CR}$ of the original $\theta =\pi /2$ pulse, we can modify the angle $\theta$ that is implemented as part of the $R_{ZX}$ gate. A more detailed description can be found at^19,20^.

In this work, by default, we choose to keep the original peak amplitude and modify the pulse duration, in order to achieve the desired $\theta$ value, as it leads to lower gate durations.

#### Pulse merging

We implement $P_{abc}(\theta _1, \theta _2)$ by merging the pulses that would constitute two separate $R_{ZX}$ gates, instead of implementing each $R_{ZX}$ gate in a serial manner, as in eq. (8), figs. 2a ,2b. We thereby reduce the gate duration by up to half. The procedure can be implemented straightforwardly, if the pulse instructions for each $R_{ZX}(\theta )$ gate are previously known and well calibrated. These can be obtained following the standard procedure in the previous Section. The $P_{abc}(\theta _1, \theta _2)$ gate implementation is as follows:

1. Schedule the two CR pulses to start running at the same time;
2. (Optional) Stretch the gate duration of the shortest CR pulse to have the same duration as the longer pulse. Simultaneously, reduce the pulse amplitude $A_\textrm{CR}$ so as to keep $\int _{t_i}^{t_f} A(t) \,\textrm{d}t$ constant. This step is not strictly required, but it was a firmware limitation present in pulse control in IBM’s quantum devices at the time the results in this work were obtained;
3. Merge the two compensation pulses. If the phase, peak amplitude, and duration of the original compensation pulses are $\phi$, $A_i, t_i$ (for the two pulses $i=1,2$), then the resulting combined compensation pulse has
  24 $$\begin{aligned} t&= \max \{t_1,t_2\}\end{aligned}$$
  25 $$\begin{aligned} A&= \frac{A_1 t_1 + A_2 t_2}{t} \end{aligned}$$

Note that we are assuming the two pulses share the same phase $\phi$, which will also be the phase of the resulting pulse, because we are working under the assumption that the Hamiltonians commute.

**Fig. 3:**
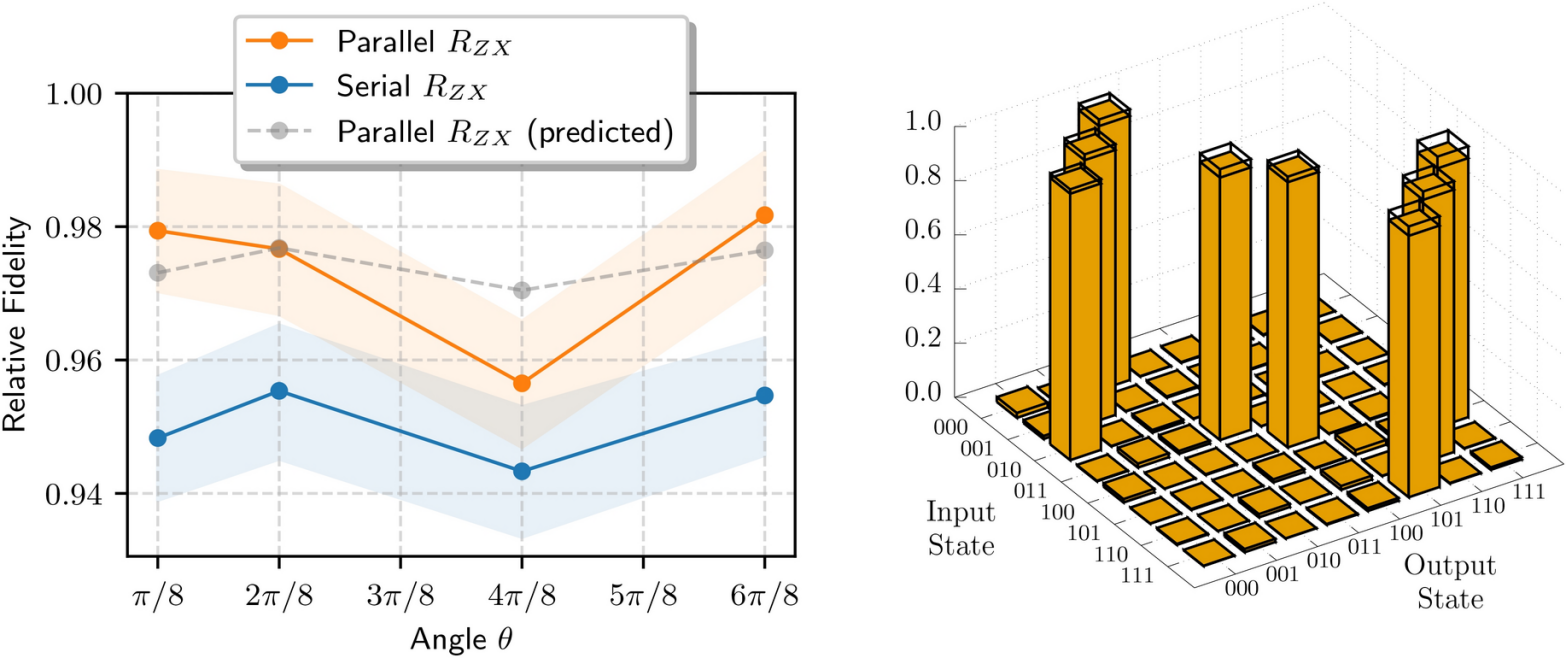
**(Left)** Measured fidelities for the parallel and serial $R_{ZX}$ gates, obtained via maximum-likelihood process tomography. The shaded regions correspond to the 90% confidence intervals. To account for SPAM errors, the fidelities are presented relative to the fidelity of the identity process ($\theta = 0$), $F_\textrm{id}=0.882\%$ (90% confidence interval: [0.876, 0.888]). In gray, we present the expected gain in fidelity that the Serial $R_{ZX}$ would have by shortening its gate duration to that of the Parallel $R_{ZX}$, using the decoherence model in eq. (26). We conclude that the Parallel $R_{ZX}$ achieves a significantly better fidelity, compatible with coherence gains due to its shortened gate time. **(Right)** As an example, we show the parallel $R_{ZX}(\pi /2)$ gate’s logical table—probability of mapping a computational basis state $\vert ijk\rangle$ into $\vert i'j'k'\rangle$—reconstructed from its process tomography. To account for SPAM errors, the truth table was normalized as described in the main text. We observe results that are close to the ideal operation (black wire frame). See Appendix E2 for a more complete characterization of the Parallel $R_{ZX}(\pi /2)$ using the Pauli Transfer Matrix.

Directly implementing $P_{abc}(\theta )$ using this procedure gives us a pulse schedule similar to that in 2a. By doing so, it is as if we are implementing the two $R_{ZX}$ gates in succession.

This is not the only way to merge the two $R_{ZX}$ gates. Here, we chose a gate duration corresponding to the duration of the longest CR pulse (that is, $t_\textrm{CR} = \max \{t_1,t_2\}$), but this duration can be chosen directly. For example, it is possible to choose a duration $\alpha t_\textrm{CR}$ ($\alpha \in \mathbb R^+$) for the CR and compensation pulses, as long as the peak amplitudes are multiplied by a factor of $1/\alpha$. As a result, the quantity $\int _{t_i}^{t_f} A(t) \,\textrm{d}t$ is unchanged, so the same $\theta$ angle is implemented. Nonetheless, since shorter durations require higher amplitudes, for sufficiently high amplitude values the physical behavior of the system is non-linear, and leads eq. (22) to no longer be valid^18^, and the implemented pulses will no longer correspond to the desired gate. In practice, there is then a trade-off between using low pulse amplitudes and low gate durations. An analysis of such a trade-off is outside the scope of this work, since here we focus on reusing the pre-calibrated gates provided by the quantum devices.

The generalization of this procedure for *n* $R_{ZX}$ gates can be found in Appendix D.

### Gate benchmarking

To demonstrate the usefulness of the procedure we described in the previous Section, we implement a parallel $R_{ZX}$ gate, $P_{abc}(\theta )$, on two pairs of qubits (*a*, *b*) and (*c*, *b*). We choose qubits $(a, b, c) = (2, 1, 3)$ of the ibmq_belem device (see Appendix A for their characterization).

In practice, $R_{ZX}$ gates on the ibmq_belem platform require echo pulses to suppress unwanted interactions. Furthermore, for ${|\theta |} > \pi / 2$, $P_{abc}(\theta )$ can be reduced to $P_{abc}(\tilde{\theta })$ with $|\tilde{\theta }|< \pi /2.$ Our implementation of $P_{abc}$ uses both of these techniques (see Appendix B).

In fig. 3 (left panel), we present the process fidelity for $P_{abc}$ (“Parallel $R_{ZX}$”) for several angles in the interval $[0, \pi ].$ The fidelities were obtained from Maximum-Likelihood Estimation (MLE) process tomography ^30^. To account for fluctuations in the calibration of ibmq_belem, we accumulated tomography data over three or more runs at different dates. For the same experiment, we calculated the MLE by pooling the data of all runs together, to reduce the effects of systematic errors of single calibrations; although this procedure in theory increases the dispersion of results, in practice it resulted in narrower confidence intervals due to more data points being used. For comparison, we also present the fidelities for the serial variant $R_{ZX}[a][b]\,R_{ZX}[c][b]$ (“Serial $R_{ZX}$”). The shaded regions correspond to the 90% confidence intervals following the procedure in Ref. ^31^. To account for State Preparation and Measurement (SPAM) errors, the fidelities are presented relative to that of the identity operation, $F_\textrm{id}=0.882$, which has a 90% confidence interval of [0.876, 0.888]. Further results can be seen in appendices E1 and E2.

We can see that the parallel $R_{ZX}$ consistently achieves a better fidelity than its serial variant. In what follows, we argue that the better performance is compatible with the gain in coherence due to the shorter gate duration.

Current quantum devices are prone to decoherence^3^, due to interactions that occur between the underlying quantum system and the macroscopic environment. As a result, when already accounting for other sources of noise, the fidelity over time is given by the exponential decay model^32^

26 $$\begin{aligned} F(t) = (1-F_0)e^{-\beta t} + F_0, \end{aligned}$$

where $\beta$ is a device-dependent constant, which is lower for less noisy devices, and $F_0 = 2^{-n}$ is the fidelity of a maximally-mixed state of *n* qubits. All else being equal, if the serial and parallel $R_{ZX}$ implementations have a duration of $t_S$ and $t_P$, respectively, then their fidelities are given by $F_S = F(t_S)$ and $F_P=F(t_P)$, respectively, from which we obtain

27 $$\begin{aligned} F_P = (1-F_0) (\frac{F_S-F_0}{1-F_0})^{t_P/t_S} + F_0. \end{aligned}$$

If $F_0$ is negligibly small (as is the case in the many-qubit setting), this expression can be simplified to

28 $$\begin{aligned} F_P = F_S^{t_P/t_S}. \end{aligned}$$

In the best case scenario, where both $R_{ZX}$ gates have the same duration, we have $t_S = 2t_P$, yielding

29 $$\begin{aligned} F_P = \sqrt{F_S}. \end{aligned}$$

When considering the $(n+1)$-qubit formulation as in eq. (13), we have $t_S = nt_P$, with which we obtain

30 $$\begin{aligned} F_P = F_S^{1/n}, \end{aligned}$$

yielding both a significant reduction in the circuit duration and a significant improvement in the gate fidelity.

In the left panel of fig. 3, we show in gray $F_S^{t_P/t_S}$, which appears to be close to the value of $F_\textrm{P}$ within the experimental error. This supports the claim that the fidelity gains we observe are compatible with a gain in coherence.

In the right panel of fig. 3 we show the reconstructed logical truth table for $P_{abc}(\pi / 2)$, that is, the probability $T_{ij}$ of obtaining a given computational basis $\vert j\rangle$ state when given as input another computational basis state $\vert i\rangle$, for $i,j=000,\ldots ,111$. To account for SPAM errors, the truth table is normalized so that its fidelity corresponds to the relative fidelity presented on the left panel, using the following procedure. Let $\mathcal S_\textrm{mle}$ be the Choi density operator of $P_{abc}(\pi /2)$ reconstructed via MLE tomography (with process fidelity $F_\textrm{mle}$), and let $S_\textrm{ideal}$ be the ideal Choi density operator of $P_{abc}(\pi /2)$. We construct the normalized operator $S_\textrm{norm}=\alpha S_\textrm{mle} + (1-\alpha ) S_\textrm{ideal}$, for $\alpha \ge 0$, such that

31 $$\begin{aligned} F(S_\textrm{norm}, S_\textrm{ideal})&:= \textrm{Tr}{S_\textrm{norm} \, S_\textrm{ideal}} \nonumber \\&= \alpha F_\textrm{mle} + (1-\alpha ) \cdot 1 \nonumber \\&= F_\textrm{mle} / F_\textrm{id} \approx 0.957. \end{aligned}$$

The bars in the figure correspond to

32 $$\begin{aligned} T^\textrm{norm}_{ij} = \textrm{Tr}{S_\textrm{norm} \, (\rho _\textrm{i}^T \otimes \rho _\textrm{j})}, \end{aligned}$$

where $\rho _i := \vert i\rangle \langle i \vert .$ The black wire frames correspond to the probabilities of the ideal process $P_{abc}(\pi /2).$

Finally, since process tomography does not discount SPAM errors, we characterize the SPAM-free performance of $P_{abc}$ using Cycle Benchmarking. Usually, one uses Interleaved Randomized Benchmarking (IRB)^33,34^, by interleaving applications of the desired operation with random Clifford gates. However, IRB scales unfavorably. For this reason, we use Pauli operators instead of Clifford, in a process called *Pauli twirling.* This results in a characterization procedure known as Cycle Benchmarking ^35^.

We use Cycle Benchmarking ^35,36^ to characterize the performance of the serial and parallel versions of $R_{ZX}(\pi /2)$, for all three-qubit Pauli channels. CB characterizes gate performance by mapping errors (both coherent, like overshooting errors, and incoherent) to errors associated with the so-called Pauli channels ^35,36^, which are then readily measurable. To perform this mapping, we first apply a multi-qubit Pauli channel, and then repeat our gate of interest several times. Between two repetitions, we apply random one-qubit Pauli operations to random qubits. We use twirling depths (gate repetitions) of $m\in \{4,8,16,32\}$; for each *m*, we generate 28 different circuits, with random Pauli operations (see Appendix E1). The output of the CB protocol are the fidelities $p_k$ associated with each Pauli channel *k*, computed by fitting the fidelity decay using the model $Ap_k^m$^17^. This allows us to isolate the error due to the gate only, and disregard the SPAM errors. As shown in Appendix E1, the average CB fidelity of the Parallel $R_{ZX}(\pi /2)$ thus obtained is $99.15(3)\%$ and that of the Serial $R_{ZX}$ is $98.16(7)\%,$ after normalizing by the fidelity of the identity process to discount for the intrinsic error of the Pauli twirling operators.

The difference in fidelities as measured with Cycle Benchmarking and MLE Tomography is explained by the facts that Cycle Benchmarking corrects for SPAM errors and does not compute a fidelity to some target gate. Cycle Benchmarking quantifies the error per interleaved Pauli, akin to IRB’s error per Clifford.

In summary, as revealed by MLE tomography, our parallelization procedure appears to systematically increase the gate fidelities, and these gains are compatible with less decoherence due to faster gate times. The only possible exception is observed for $P_{abc}(\pi /2)$, whose fidelity is slightly lower than expected. Given that this is the gate with the longest time, it is possible that it accumulated coherent errors that become measurable. Cycle Benchmarking reinforces the idea that shorter gate times are the reason for higher fidelities: unlike tomography, CB does not measure fidelity to some target gate, but we still observe gains in fidelity. Because CB discounts SPAM errors, the high CB fidelities also suggest that SPAM is a significant source of error. This is in line with the observation that the MLE fidelity of the identity operation is relatively low (approximately 88%), and justifies the normalization procedure in fig. 3.

## Applications

So far, we have showed how a pulse-parallelized version of $R_{ZX}$ gates can be beneficial for the simulation of Hamiltonians given by eq. (12). In this Section, we show that, by extending the parallelization technique to CNOT and CZ gates, we can extend its use to simulating more general Hamiltonians, and implementing oracles.

### Parallelized CNOT gates

**Fig. 4:**
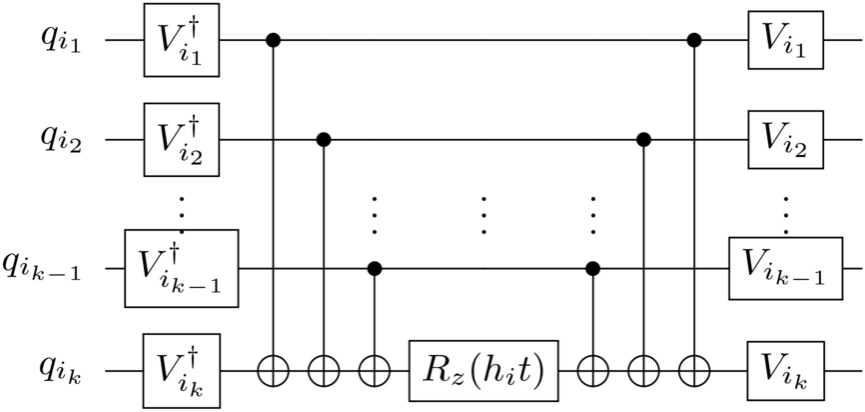
Time evolution of the Pauli term $h_i O_{i_1} \cdots O_{i_k}$ using CNOT gates. We use qubit $i_k$ as the common CNOT target qubit, but any of the other $k-1$ qubits could be used.

**Fig. 5:**
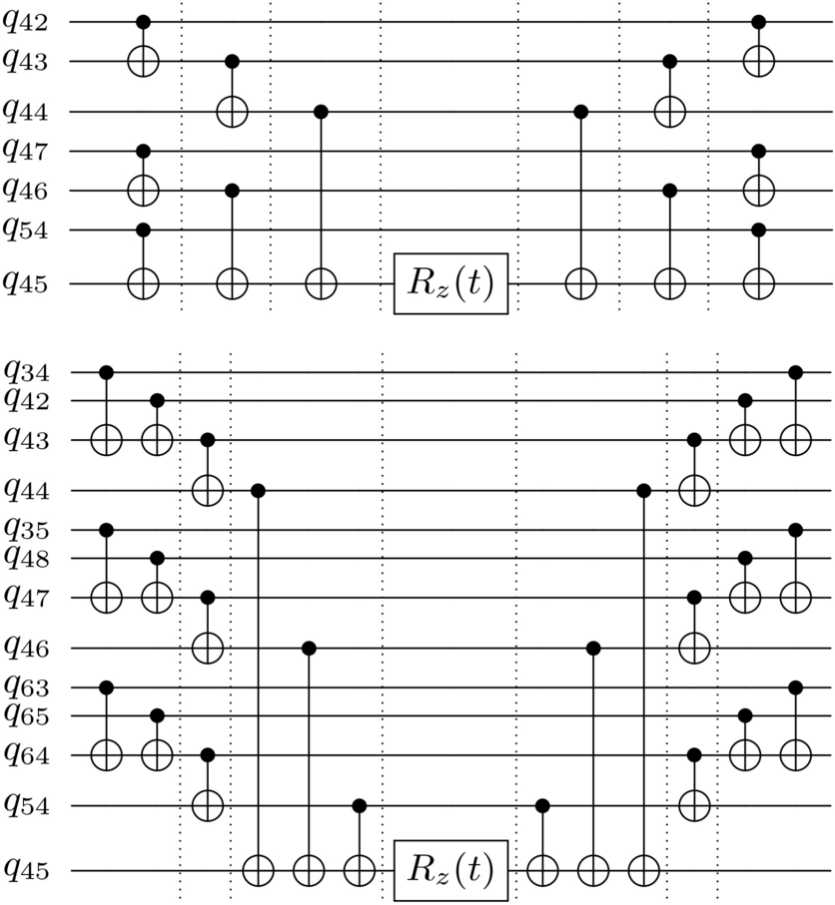
Hamiltonian evolution of a Pauli term of the form $ZZ\ldots Z$. The qubit indices mark possible physical qubits in the ibm_brisbane device that would enable these circuits to be directly implemented, without connectivity constraints. The dotted lines mark one time step $\Delta t$, for a total duration of $6\delta t$. We discount the $R_z$ gate, whose implementation is virtual and considered instantaneous. As an example, if the ibm_brisbane device’s decoherence is such that only circuits of duration $6\Delta t$ can be implemented with high fidelity, then **(Top)** using traditional techniques enables us to implement, at most, the 7-qubit term $\exp (-i t Z_1\ldots Z_7)$. **(Bottom)** However, using the parallelization techniques introduced in this work, for the same circuit duration, we can instead implement a 13-qubit term, thereby significantly extending the utility of low-depth circuits on current quantum devices.

**Fig. 6:**
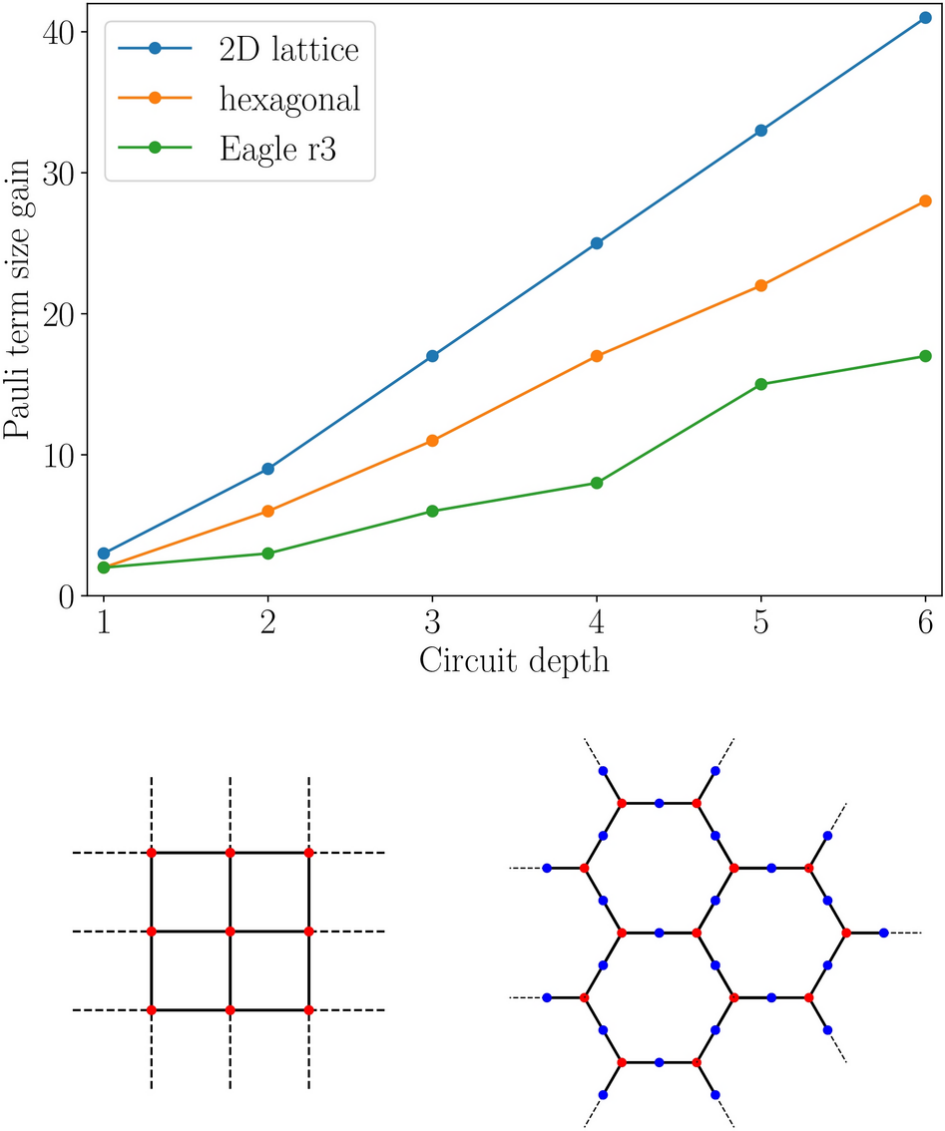
Parallelization benefit for different device layouts. **(Top)** Increase in the maximum qubit size of the simulatable Pauli-terms, when employing CNOT parallelization, assuming a fixed circuit depth. For example, in fig. 5, for depth 3, the simulatable Pauli term goes from being 7-qubit to 13-qubit in size, gaining 6 qubits. Among the layouts tested, for a 2D lattice configuration **(bottom left)**, the benefit is greatest, and it increases linearly with the circuit depth used. Configurations with weaker qubit connectivities, such as **(bottom right)** those of a standard hexagonal layouts (red dots stand for qubits) or those like IBM’s Eagle r3 processor types (both red and blue dots stand for qubits) yield smaller, but still significant improvements. IBM’s ibm_brisbane possesses the latter layout.

Using a similar approach to the $P_{abc}$ implementation, and eq. (21), we may apply several CNOT gates in parallel, at the pulse level. For $(n+1)$ qubits, we have

33 $$\begin{aligned} \prod _{c \in C} \textrm{CNOT}_{ct}&= P_{t,{\textbf {c}}}(\pi /2) (Q_t S_{c_1}^\dagger \cdots S_{c_n}^\dagger ) \end{aligned}$$

where $Q = X^{3n/2 \text { mod } 2}$, $C = \{c_1,\ldots , c_n\}$ is the set of *n* control qubits, and there is a common target qubit *t*. For the generalized version with common control qubits, see Appendix C.

This type of parallel CNOT gate implementation proves particularly useful for Hamiltonian simulation. The evolution of an individual Pauli term $h_i O_{i_1} \cdots O_{i_k}$ (with $O \in \{X,Y,Z\}$) can be implemented by first mapping it to the term $h_i Z_{i_1} \cdots Z_{i_k}$ through a basis change on each qubit *j* (as in eq. (4)) and then applying the evolution for that term (see fig. 4).

Following this procedure, the evolution of a Pauli term may be performed as in fig. 4, which relies on the application of many CNOT gates with the same target.

A possible strategy is then to evolve the $\textrm{CNOT}$ gates in parallel. The main idea is that the $\textrm{CNOT}$s may be written as a parallelization of $R_{ZX}$ gates.

If the Pauli term of interest has $n+1$ components that are not the identity, then its evolution would require *n* CNOT gates in succession before and after the $R_z$ gate. If the quantum device being used is such that all these CNOT gates share the same target at the physical level, then the duration of the CNOT gate implementation could be reduced by a factor of 1/*n*, and the circuit fidelity significantly improved (see section 3.3).

In practice, it is uncommon for current quantum devices, and especially those based on transmon qubits, to have the high qubit connectivities that would enable these significant improvements. For quantum devices based on superconducting qubits, each qubit can usually only be directly entangled with a small number of neighboring qubits. Entanglement to more distant qubits needs to be indirectly applied, through the use of SWAP gates. Current quantum devices tend to have qubits arranged on a lattice, with each at most directly connected to 3 neighboring qubits. More often, each qubit is only connected to 1 or 2 neighbors. As a result, for most situations, we can expect only 2 or 3 CNOT gates to be parallelized at the pulse level at a time.

Nonetheless, as can be seen in fig. 5, even with this practical limitation, CNOT gate parallelization can yield significant improvements. While the setup in fig. 4 is the most straightforward way to implement a Pauli term evolution, there are other valid CNOT gate arrangements that result in valid circuits^37^. In general, if we wish to implement a Pauli term with *n* non-identity components, we only need to ensure that the CNOT gates apply the gate $X^\alpha$ to the qubit with the $R_z$ gate, with $\alpha$ equal to the parity of the *n* qubits with the *n* non-identity components.

Without connectivity limitations, the optimal CNOT gate configuration permits CNOT gates applied with a circuit depth of *d* to implement a Pauli term with $n=2^d$. However, as previously mentioned, current connectivity constraints lead the practical implementation to be far from optimal. For example, for the ibm_brisbane device, a depth of 3 for the CNOT gate portion only allows the simulation of, at most, a 7-qubit Pauli term, and not 8, since the optimal implementation would require a qubit directly connected to 4 neighbors (see fig. 5). Nonetheless, using the parallelization techniques in our work, we increase the complexity of the simulatable Pauli terms, enabling the possibility of implementing a 13-qubit Pauli term using the same circuit depth. In essence, we gain access to 6 additional qubits for a fixed-depth simulation.

In general, the benefit gained from parallelizing CNOT gates will depend on the qubit connectivity layout of the qubit device. For a given depth *d*, layouts with more qubit-to-qubit connections will observe greater improvements in the number of additional qubits that can be included in the simulation, when parallelization is applied. See fig. 6 for an analysis of some common layouts.

### Parallelized CZ gates

Similarly, we may implement CZ gates in parallel, by relying on the parallel CNOT setup in eq. (33), yielding the implementation

34 $$\begin{aligned} \prod _{c \in C}\textrm{CZ}_{ct}&= H_{t} P_{t,{\textbf {c}}}(\pi /2) (Q_t H_t S_{c_1}^\dagger \cdots S_{c_n}^\dagger ), \end{aligned}$$

where *H* stands for the Hadamard gate. Note that, since CZ is symmetric with respect to the 2 qubits it is applied to, here *t* simply stands for the index of the shared qubit among the parallel CZ gates.

**Fig. 7:**
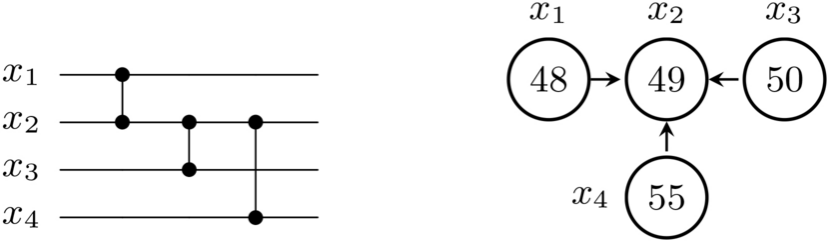
**(Left)** Quantum circuit that implements the phase oracle $U = I - 2\sum _{i\in \iota _f}\vert i\rangle \langle i \vert$, where *i* is a bitstring and $i\in \iota _f$ iff $f(i)=1$, with $f(i)=f(x_1x_2x_3x_4)=x_1x_2+x_2x_3+x_2x_4$ mod 2. Different functions *f* would lead to different placements for the CZ gates. In this case, since they share a common qubit ($x_2$), they can be parallelized at the pulse level. **(Right)** Possible implementation of the phase oracle in the ibm_brisbane device, using physical qubits 48, 49, 50, and 55 to encode the variables $x_1$ to $x_4$, respectively. The arrows indicate the direction under which a direct $R_{ZX}$ implementation is possible, with the arrow start (resp. end) corresponding to the *Z* (resp. *X*) operator. Other qubit connections in ibm_brisbane not relevant for the oracle implementation are not presented.

Among other uses, parallelizing CZ gates could prove useful to speed up the implementation of amplitude amplification. For example, a phase oracle whose encoded function corresponds to a Boolean formula, that in disjunctive normal form is a sum of terms of 2 variables, can be entirely implemented with CZ gates. See an oracle example in fig. 7.

**Fig. 8:**
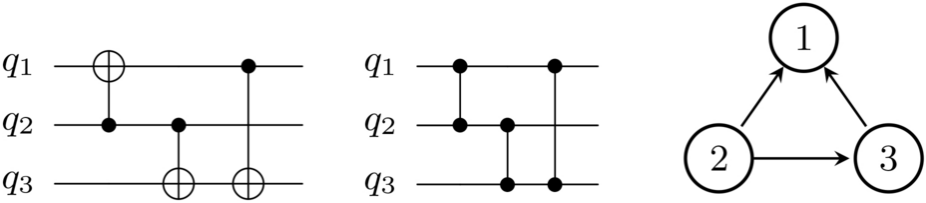
**(Left)** With CNOT gates, the parallelization technique can only be applied to 2 of the CNOT gates if either the physical qubit $q_3$ (common target, ${2}^{\textrm{nd}}$ and ${3}^{\textrm{rd}}$ gates) or $q_2$ (common control, ${1}^{\textrm{st}}$ and ${2}^{\textrm{nd}}$ gates, using technique in Appendix C) can be used as a common target for two $R_{ZX}$ gates. Unfortunately, the parallelization technique is not applicable for some device layouts **(right)**, since then only configurations with a common target in $q_1$ are possible. **(Middle)** For CZ gates, the parallelization technique can be applied in theory with any qubit as the shared one and, for this particular example, to qubit $q_1$, thereby parallelizing the ${1}^{\textrm{st}}$ and ${3}^{\textrm{rd}}$ gates.

The conclusions previously reached for the parallelization of CNOT gates mostly apply to CZ gates as well. The benefit of parallelizing CZ gates is greater the higher the qubit-to-qubit connectivity. However, unlike CNOT gates, CZ gates are symmetric, allowing more freedom to implement a desired circuit efficiently at the pulse level (see fig. 8 for an example).

## Discussion

In this work, we presented a pulse parallelization technique that is readily available in NISQ devices. Using IBM’s ibmq_belem device, we demonstrated that the parallelization of $R_{ZX}$ gates yields a fidelity advantage over their serial counterparts. As measured using Cycle Benchmarking, the minimum fidelity increase gained from parallelization was from 98.16(7)% to 99.15(3)%, for the Parallel $R_{ZX}(\pi /2)$.

As the required pulses are straightforward to implement in most superconducting qubit architectures, including cloud-based-processors, the proposed approach has the potential to enable the simulation of larger circuits with higher fidelities, in NISQ devices. In particular, it seems promising for Hamiltonian simulation, as many quantum simulation problems can be readily decomposed in terms of $R_{ZX}$ gates. Some well-known circuits, such as the encoding of error-correction codes ^6^, can also benefit from the parallelization of many CNOT and CZ gates. CNOT gates with a common target can be thought of as multi-qubit parity gates, which have been recently analyzed in Ref. ^26^, both by deriving the theoretical system Hamiltonian and by confirming the gate behavior experimentally.

Different opportunities for further work present themselves. First, in our demonstration, we did not exploit the freedom to shorten the gate times. We focused on the simplest approach—to superpose the pulses as provided by the CNOT callibration—but it would be interesting to find out the minimal gate times before non-linear effects become relevant. Second, the parallelization could be tried in different architectures, like ion traps or cold atoms, which have different native Hamiltonians. Third, while we focused on $R_{ZX}$ gates with shared target due to echo limitations, it would be interesting to see whether sharing the control qubit is feasible, or if one could implement parallel CZ gates. Finally, one could look into experimentally assessing the fidelity of many-qubit parallelization, starting with 3 parallel gates (see fig. 6, 7 for examples available to current quantum devices).

Going beyond these generalizations, one could investigate the generation of arbitrary 3-qubit gates via either variational processes as outlined in^17^ or via KAK decomposition^23,38–42^, and analyze if the additional freedom attained by controlling the pulses directly is useful.

In conclusion, our pulse parallelization technique demonstrates a path to higher fidelities and faster gate times on NISQ devices, with potential applications to quantum simulation, error correction, and broader quantum computation. Further work could focus on optimizing and generalizing this technique across different qubit architectures and larger qubit counts.

## Supplementary Information

Supplementary Information.

## Data Availability

Data sets generated during the current study are available from the corresponding author on request.
