# Supplementary material for: Native multi-qubit gates in transmon qubits via synchronous driving
Source: Sci Rep. 2024 Oct 29;14:26042. doi: 10.1038/s41598-024-76396-9 (PMC11522314; doi:10.1038/s41598-024-76396-9)
Supplement: Supplementary file 1 — Supplementary Information. [file 41598_2024_76396_MOESM1_ESM.pdf]

Supplemental Material
Native multi-qubit gates in transmon qubits via synchronous driving

Sagar Silva Pratapsi,[1,2,*] Diogo Cruz,[1,2,*] and Paulo André[1,2]
[1]Instituto Superior Técnico, Universidade de Lisboa, Portugal
[2]Instituto de Telecomunicações, Portugal

Quantum computation holds the promise of solving computational problems which are believed to be classically intractable. However, in practice, quantum devices are still limited by their relatively short coherence times and imperfect circuit-hardware mapping. In this work, we present the parallelization of pre-calibrated pulses at the hardware level as an easy-to-implement strategy to optimize quantum gates. Focusing on R_{ZX} gates, we demonstrate that such parallelization leads to improved fidelity and gate time reduction, when compared to serial concatenation. As measured by Cycle Benchmarking and Process Tomography, we reduce gate errors by half. We show that this strategy can be applied to other gates like the CNOT and CZ, and it may benefit tasks such as Hamiltonian simulation problems, amplitude amplification, and error-correction codes.

	Qubits		
**Property**	**2**	**1**	**3**
T_1 (μs)	69.3	78.0	53.6
T_2 (μs)	38.6	63.8	56.5
frequency (GHz)	5.36	5.25	5.17
anharmonicity (GHz)	-0.33	-0.32	-0.34
readout error (%)	2.4	3.2	3.4
$p(1\|0)$ (%)	3.76	5.50	5.78
$p(0\|1)$ (%)	1.04	0.90	1.02
readout length (ns)	5351.1	5351.1	5351.1
$\sqrt{X}$ infidelity (%)	0.030	0.041	0.067
$\sqrt{X}$ duration (ns)	35.6	35.6	35.6
CNOT(2,1) infidelity (%)		1.1	
CNOT(2,1) duration (ns)		412.4	
CNOT(3,1) infidelity (%)		1.4	
CNOT(3,1) duration (ns)		398.2	

TABLE I. Device configuration.

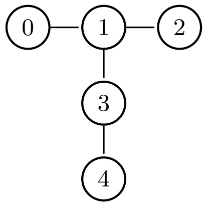

FIG. 1. Device layout. The nodes represent the qubits, and their labels, while the edges indicate the possible direct CNOT gate implementations.

Appendix A: Device information

The observed results were obtained on IBM's `ibmq_belem` device, in backend version 1.0.35. Qubits (2, 1, 3) were used, respectively, as (control, target, control), to obtain the tomography results in Fig. 4 of the main text and figs. 3 to 5 of the Supplemental Material. Its layout and a representative configuration are displayed in fig. 1 and table I, respectively. We note that, due to limitations in the number of circuits allowed to run concurrently in IBM's devices, and the large number of circuits required to obtain these results, IBM's device may have been recalibrated between some of the runs. Nonetheless, the device configuration did not deviate significantly from the values shown in table I.

Appendix B: Pulse-level optimizations

In Fig. 2 of the main text, we showed two possible pulse implementations for the concatenation of two R_{ZX} gates (top panel) and the parallel variant P_{abc} in `ibmq_belem` (bottom panel). For the purposes of this section, we will refer to these pulse implementations as $\dot{R}_{ZX}$ and $\dot{P}_{abc}$, respectively.

Unfortunately, in practice, the error associated with $\dot{R}_{Z_aX_b}$ and $\dot{P}_{abc}$ is high, so we use two techniques to reduce their error: angle reduction and echo sequences. This results in the gate implementation

$$\begin{aligned} R_{Z_aX_b}(\theta) &:= G_{ab}(\theta)\dot{R}^{\text{echo}}_{Z_aX_b}(\tilde{\theta}) \\ P_{abc}(\theta) &:= G_{b,(a,c)}(\theta)\dot{P}^{\text{echo}}_{abc}(\tilde{\theta}), \end{aligned} \tag{B1}$$

As explained in appendix B 1, the mapping $\theta \to \tilde{\theta}$ allows us to bring the angle of R_{ZX} to the range $[-\pi/2, \pi/2]$. This mapping requires using the additional gates G. On the other hand, $\dot{R}^{\text{echo}}_{ZX}$ and $\dot{P}^{\text{echo}}_{abc}$ refer to the echoed variants, defined in appendix B 2.

In fig. 2, we show the actual pulse sequences that we used for the serial and parallel versions of $P_{abc}(\pi/2)$, using the decomposition in eq. (B1).

1. Angle reduction

In the default implementation of $R_{ZX}(\theta)$ gates in IBM's devices, the duration of the CR pulse and compensation tones increases proportionally to the angle θ of the gate. As a result, for high values of θ, the gate duration is substantial, leading to noticeable state decoherence. Moreover, the basis gates for the device are calibrated based on their performance for low θ (in particular, $\theta = \pm\pi/4$ for the CNOT gate with echo), resulting in an accumulation of coherent errors

* Equal contribution.

for the uncalibrated high θ values. To circumvent this limitation, it is possible to construct the R_{ZX} (and $P_{t,\mathbf{c}}$) gate for any θ value using only the pulse construction for $|\theta| \leq \pi/2$, without echo, or $|\theta| \leq \pi/4$, with echo.

Since the R_{ZX} gate is periodic, so that $R_{ZX}(\theta) = R_{ZX}(\theta + 2\pi)$, for the sake of simplicity, we consider that the input angle $\theta \in \mathbb{R}$ is pre-mapped to the range $(-\pi, \pi]$.

Let us map θ to the range $[-\pi/2, \pi/2]$ range using the function

$$\tilde{\theta} := \begin{cases} \theta - \operatorname{sign}(\theta)\pi, & \text{if } \frac{\pi}{2} < |\theta| \leq \pi, \\ \theta, & \text{if } |\theta| \leq \frac{\pi}{2} \end{cases} \tag{B2}$$

$$\tilde{\boldsymbol{\theta}} := (\tilde{\theta}_1, \ldots, \tilde{\theta}_n). \tag{B3}$$

We can then reduce $R_{ZX}(\theta)$ to $R_{ZX}(\tilde{\theta})$ by appending a correction gate G, because

$$R_{Z_a X_b}(\theta) = G_{ab}(\theta)\, R_{Z_a X_b}(\tilde{\theta}), \tag{B4}$$

where

$$G_{ab}(\theta) := \begin{cases} -i \operatorname{sign}(\theta)(Z_a X_b), & \text{if } \frac{\pi}{2} < |\theta| \leq \pi \\ I_a I_b, & \text{if } |\theta| \leq \frac{\pi}{2}, \end{cases} \tag{B5}$$

so that we may only use angles $|\tilde{\theta}| \leq \frac{\pi}{2}$ in R_{ZX}. The $-i \operatorname{sign}(\theta)$ factor corresponds to a global phase and can be disregarded. Depending on the θ value, the additional gates $Z_a X_b$ may need to be applied.

Similarly, we can reduce the angle of $P_{t,\mathbf{c}}(\boldsymbol{\theta})$, since it is a concatenation of $R_{Z_{c_i} X_t}(\theta_i)$ gates,

$$P_{t,\mathbf{c}}(\boldsymbol{\theta}) = G_{t,\mathbf{c}}(\theta) P_{t,\mathbf{c}}(\tilde{\boldsymbol{\theta}}), \tag{B6}$$

where

$$G_{t,\mathbf{c}}(\theta) := G_{c_1 t}(\theta) \cdots G_{c_n t}(\theta) \tag{B7}$$

$$= Q^Z_{\mathbf{c}}(\boldsymbol{\theta})\, Q^X_t(\boldsymbol{\theta}). \tag{B8}$$

In the last equality we separated the Z and X gates, since they simplify to

$$Q^Z_{\mathbf{c}}(\boldsymbol{\theta}) := (-i \operatorname{sign}(\theta))^{\Delta(\boldsymbol{\theta})} Z_{c_1}^{\delta(\theta_1)} \cdots Z_{c_n}^{\delta(\theta_n)} \tag{B9}$$

$$Q^X_t(\boldsymbol{\theta}) := X_t^{\Delta(\boldsymbol{\theta}) \bmod 2}, \tag{B10}$$

where we made use of the definitions

$$\delta(\theta) := \begin{cases} 1, & \text{if } \frac{\pi}{2} < |\theta| \leq \pi \\ 0, & \text{if } |\theta| \leq \frac{\pi}{2} \end{cases} \tag{B11}$$

$$\Delta(\boldsymbol{\theta}) := \sum_i \delta(\theta_i). \tag{B12}$$

The $(-i \operatorname{sign}(\theta))^{\Delta(\boldsymbol{\theta})}$ factor corresponds to a global phase and can be disregarded. When the θ angles are identical, the expression simplifies to

$$P_{t,\mathbf{c}}(\theta) = Q^Z_{\mathbf{c}}(\theta)\, Q^X_t(\theta)\, P_{t,\mathbf{c}}(\tilde{\theta}) \tag{B13}$$

with

$$\Delta(\theta) := n\delta(\theta) \tag{B14}$$

$$Q^Z_{\mathbf{c}}(\theta) := (Z_{c_1} \cdots Z_{c_n})^{\delta(\theta)} \tag{B15}$$

$$Q^X_t(\theta) := X_t^{\Delta(\theta) \bmod 2}. \tag{B16}$$

For even n, $Q^X_t(\theta)$ is always the identity, so no additional X gates are applied. Moreover, note that, in general, the implementation of the Z gates is virtual, so it does not constitute an additional source of error.

2. Echo sequences

Echo sequences are a basis-change technique to mitigate coherent errors [1–5]. We define echoed sequence for the R_{ZX} gate as

$$R^{\text{echo}}_{Z_a X_b}(\theta) := X_a R_{Z_a X_b}(-\tfrac{\theta}{2}) X_a R_{Z_a X_b}(\tfrac{\theta}{2}). \tag{B17}$$

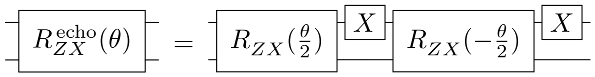

For the serial double version of the R_{ZX} gate, we apply the echo sequence to each gate separately,

$$R^{\text{echo}}_{Z_c X_b}(\theta) R^{\text{echo}}_{Z_a X_b}(\theta) \tag{B18}$$

$$= X_c R_{Z_c X_b}(-\tfrac{\theta}{2}) X_c R_{Z_c X_b}(\tfrac{\theta}{2})$$
$$X_a R_{Z_a X_b}(-\tfrac{\theta}{2}) X_a R_{Z_a X_b}(\tfrac{\theta}{2}), \tag{B19}$$

which is the gate we use in our implementation. By commuting the two middle R_{ZX} gates, and some X gates, we may also define an echoed version of the parallel variant. We obtain

$$(X_a X_c) R_{Z_c X_b}(-\tfrac{\theta}{2}) R_{Z_a X_b}(-\tfrac{\theta}{2})$$
$$(X_a X_c) R_{Z_c X_b}(\tfrac{\theta}{2}) R_{Z_a X_b}(\tfrac{\theta}{2}), \tag{B20}$$

yielding

$$P^{\text{echo}}_{abc}(\theta) := (X_a X_c) P_{abc}(-\tfrac{\theta}{2})(X_a X_c) P_{abc}(\tfrac{\theta}{2}) \tag{B21}$$

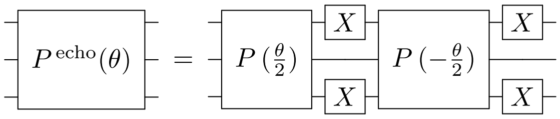

An example of the pulses associated with these echoed gates can be seen in fig. 2.

The echoed implementation of the parallel gate generalizes to n qubits and different θ angles, yielding

$$P^{\text{echo}}_{t,\mathbf{c}}(\boldsymbol{\theta}) := X_{\mathbf{c}} P_{t,\mathbf{c}}(-\tfrac{\boldsymbol{\theta}}{2}) X_{\mathbf{c}} P_{t,\mathbf{c}}(\tfrac{\boldsymbol{\theta}}{2}), \tag{B22}$$

where $X_{\mathbf{c}}$ denotes the application of X gates to all $(n-1)$ control qubits.

Appendix C: Parallel R_{ZX} gates with common control qubits

In this work, we have focused on a parallelized version of the R_{ZX} gate where the target qubit is shared, yielding the gate in Eq. (13) of the main text. Using a similar reasoning, it is possible to define the gate

$$P_{c,\mathbf{t}}(\boldsymbol{\theta}) := \exp\left(-i \sum_{i=1}^{n} \frac{\theta_i}{2} Z_c X_{t_i}\right), \tag{C1}$$

$$\text{with } \mathbf{t} = (t_1, \ldots, t_n), \tag{C2}$$

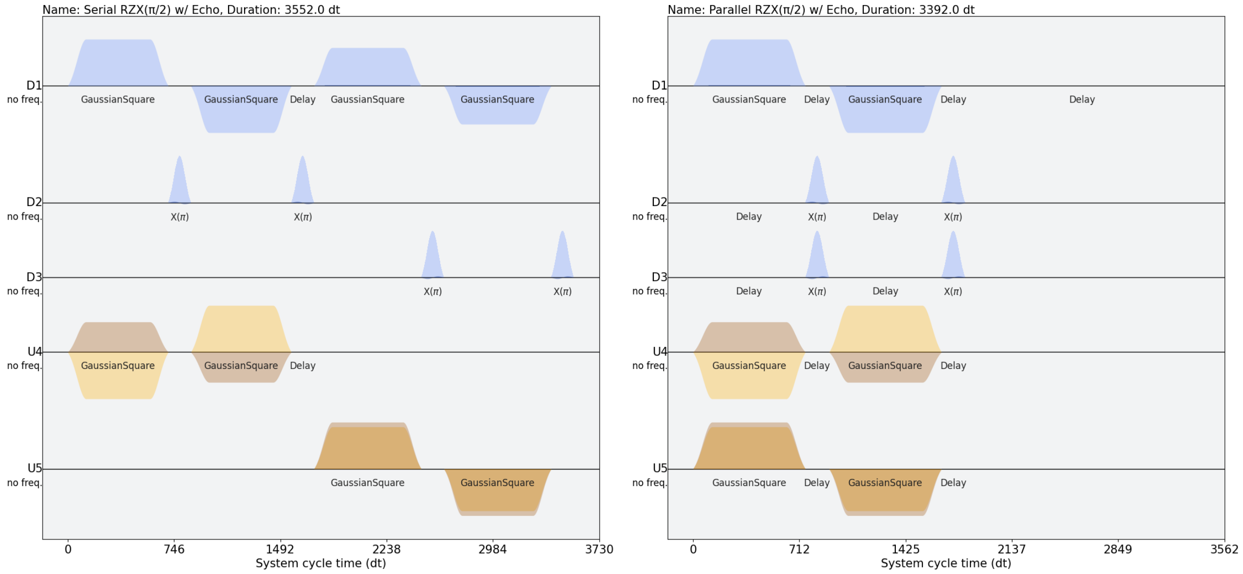

FIG. 2. Example pulse sequence for the *echoed* serial **(left)** and parallel **(right)** versions of the double $R_{ZX}(\pi/2)$ gate, for the `ibmq_belem` device, as described in appendix B. As with the unechoed version (c.f. Fig. 2 of the main text, the gate duration can be reduced by up to half by using the parallel version. For $\theta = \pi/2$, the gate G in eq. (B7) is equal to the identity operation.

where n R_{ZX} gates use a shared control qubit and distinct target qubits. Under this approach, the CR pulse parallelization would proceed as previously described, but there would be no need for merging compensation pulses, since each of these pulses would be applied to a separate target qubit.

Unfortunately, this approach does not prove as fruitful as the one with the common target qubit, since the reasoning behind the application of echo, as explained in appendix B 2, no longer holds. Consequently, suppressing some of the undesired Pauli terms in the CR Hamiltonian becomes non-trivial, and the naïve pulse parallelization achieves poor fidelity.

Nonetheless, a high-fidelity implementation can be done by using the common-target gate, as given by

$$\tilde{P}_{c,\mathbf{t}}(\boldsymbol{\theta}) = H_c H_{\mathbf{t}} P_{c,\mathbf{t}}(\boldsymbol{\theta}) H_c H_{\mathbf{t}}, \tag{C3}$$

which can take advantage of the echo implementation of the $P_{t,\mathbf{c}}(\boldsymbol{\theta})$ gate.

If we consider the most general case

$$\hat{P}_{\mathbf{c},\mathbf{t}}(\boldsymbol{\theta}) := \exp\left(-i\sum_{i=1}^{n}\frac{\theta_i}{2}Z_{c_i}X_{t_i}\right), \tag{C4}$$

$$\text{with } \mathbf{t} = (t_1,\ldots,t_n), \quad \mathbf{c} = (c_1,\ldots,c_n), \tag{C5}$$

we run into similar issues as before, since there is not a straightforward way to implement echo for the terms that share control qubits. Moreover, we may also have qubits that act both as a control and a target, depending on the Pauli term. In general, such components are not possible to parallelize, as they can be used to build gates that cannot be implemented with a single CR pulse.

In order to still take advantage of parallelization in this setting, a suboptimal but simple method is to partition the $\hat{P}_{\mathbf{c},\mathbf{t}}$ gate into a product of P and $\tilde{P}$ gates, and parallelize each of these gates separately. It may be possible to improve on this method by relying on more complex circuit optimization techniques, but these are outside the scope of this work.

Given the straightforward connection between R_{ZX} gates and CNOT and CZ gates, the same principles can be applied to parallelize CNOT and CZ gates, when the gates have some control qubits in common.

Appendix D: Pulse parallelization for n R_{ZX} gates

It is straightforward to generalize the procedure in Section III.B.2 of the main text in order to parallelize n R_{ZX} gates:

1. Schedule the n CR pulses to start running at the same time.

2. (Optional) Stretch the gate duration of the shorter CR pulses to have the same duration as the longest pulse. Simultaneously, reduce the pulse amplitude A_{CR} so as to keep $\int_{t_i}^{t_f} A(t)\,\mathrm{d}t$ constant.

3. Merge the n compensation pulses. If n is large, the computed amplitude may be higher than the maximum amplitude $A_{\max}$ below which Eq. (22) of the main text stills holds, so the gate duration may need to be increased to compensate. If the phase, peak amplitude, and duration of the original compensation pulses are ϕ, A_i, t_i (for the n pulses $i \in \{1,\ldots,n\}$), then the resulting com-

bined compensation pulse has parameters

$$S := \sum_i A_i t_i \tag{D1}$$

$$t = \max\left\{S/A_{\max}, \max_i t_i\right\} \tag{D2}$$

$$A = \min\left\{A_{\max}, \frac{S}{t}\right\} \tag{D3}$$

Note that we assumed that the pulses all have equal phases ϕ, following our assumption that their corresponding Hamiltonians commute.

Appendix E: Characterization of parallel implementation

To better characterize our implementation of the parallel gate P_{abc}, in this section we present a more complete experimental characterization of $P_{abc}(\pi/2)$. We do this because $P_{abc}(\pi/2)$ is a Clifford operator, so we can apply Cycle Benchmarking to characterize its fidelity (see the following sub-section). As we can see from Fig. 3 of the main text, this is also the gate that presents the highest errors, so it serves as Done. We updated the main text with a short comment, and also the Appendices.a lower bound for the other angles.

1. Cycle Benchmarking

As mentioned in the main text, we ran Cycle Benchmarking (CB) [6, 7] for all three-qubit channels. We ran CB sequences by interspersing m layers of random Pauli operators, with $m \in \{4, 8, 16, 32\}$. The values of m are called the *twirling depths*. For each depth, we generated 28 different sets of random Pauli operators; we call each of these 28 sets a *sample*. The fidelity p_k associated with each Pauli channel k is extracted via a fit, assuming that the measured fidelity decays as Ap_k^m [1]. This enables us to isolate the gate fidelity, and disregard the state-preparation-and-measurement (SPAM) errors. As the computed fidelities include the error contributions from Pauli-twirling, we also run the same circuits but without the tested gate, to compute the fidelity $p_k^{(\mathrm{ref})}$ associated with the Pauli-twirling component alone. Consequently, the resulting gate fidelities after deducting the SPAM and Pauli-twirling errors are given by $p_k/p_k^{(\mathrm{ref})}$. The obtained fidelities are shown in fig. 3.

2. Pauli Transfer Matrix

We provide a visualization of our implementation of the parallel R_{ZX} operation, P_{abc}, for the angle $\theta = \pi/2$, by reconstructing its Pauli Transfer Matrix (PTM). To do so, we begin with the reconstruction from Maximum-Likelihood Estimation (MLE) Process Tomography [8], and subsequently calculate the PTM.

Although quantum processes are in general operators between complex vector spaces, it is possible to fully describe them with a real matrix whose elements belong to the real interval $[-1, 1]$, using the so-called *Pauli Transfer Matrix* [9]. The elements of the Pauli Transfer Matrix T, associated with the quantum process Λ, are given by

$$T_{ij} = \mathrm{Tr}\{P_j\,\Lambda(P_i)\},$$

where P_i and P_j are the Pauli strings taken from $\{I, X, Y, Z\}^{\otimes 3}$, ordered lexicographically. Here, I is the 2×2 identity and X, Y, Z are the Pauli operators. The PTM of our three-qubit process is therefore a $4^3 \times 4^3 = 64 \times 64$ real matrix.

From the Pauli Transfer Matrix, we can infer several properties of the operator Λ. The trace-preserving condition is met if and only if the first row is 1 followed by all zeroes, i.e., $T_{0j} = \delta_{0j}$. If Λ is unital (as unitary matrices are), then the first column must also be 1 followed by all zeroes. The PTMs T_χ of general quantum processes χ also multiply, that is, $T_{\Lambda\circ\chi} = T_\Lambda T_\chi$. For more information on the PTM see, for example, Ref. [9].

Having reconstructed the operator Λ for the gate $P_{abc}(\pi/2)$ via maximum-likelihood tomography, we can calculate the PTM, which we present in fig. 4. In fig. 5 we show the difference between the PTM of our implementation of $P_{abc}(\pi/2)$ and its ideal PTM. The most significant finding, especially in fig. 5, is that the difference to the ideal process reveals a structure similar to the PTM itself. This happens because we have not corrected for SPAM errors, so there is a large difference between the peak values of the PTM and their ideal value of ± 1. Nonetheless, the fidelity reconstructed from this PTM matrix (done indirectly via the Choi matrix) is enough to show the parallelization yields an advantage over serialization, as can be seen in Fig. 3 of the main text.

[1] Y. Kim, A. Morvan, L. B. Nguyen, R. K. Naik, C. Jünger, L. Chen, J. M. Kreikebaum, D. I. Santiago, and I. Siddiqi, Nature Physics **18**, 783 (2022), arXiv:2108.10288 [quant-ph].

[2] N. Sundaresan, I. Lauer, E. Pritchett, E. Magesan, P. Jurcevic, and J. M. Gambetta, PRX Quantum **1**, 020318 (2020).

[3] T. Alexander, N. Kanazawa, D. J. Egger, L. Capelluto, C. J. Wood, A. Javadi-Abhari, and D. C McKay, Quantum Science and Technology **5**, 044006 (2020).

[4] L. B. Nguyen, G. Koolstra, Y. Kim, A. Morvan, T. Chistolini, S. Singh, K. N. Nesterov, C. Jünger, L. Chen, Z. Pedramrazi, B. K. Mitchell, J. M. Kreikebaum, S. Puri, D. I. Santiago, and I. Siddiqi, PRX Quantum **3**, 037001 (2022).

[5] M. M. Ibrahim, H. Mohammadbagherpoor, C. Rios,

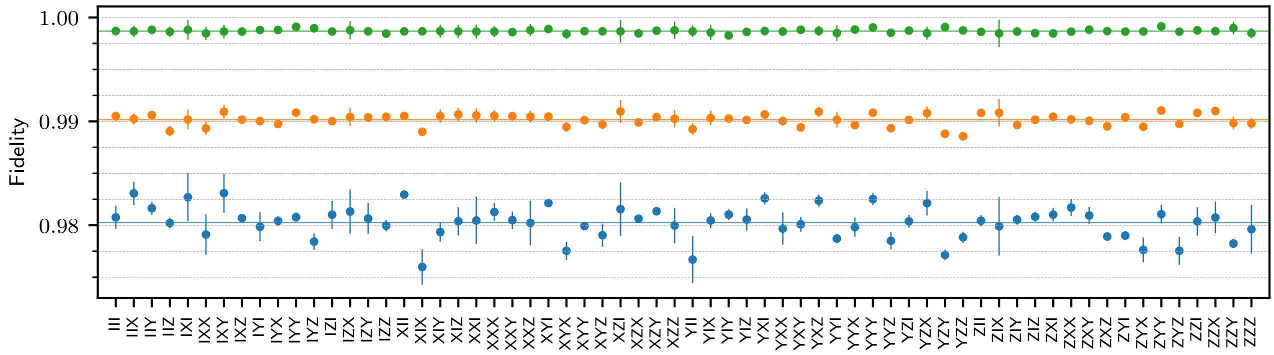

FIG. 3. Fidelities obtained from Cycle Benchmarking for the identity (green) and the parallel (orange) and serial (blue) version of two $R_{ZX}(\pi/2)$ gates, for all the different Pauli channels. The horizontal lines correspond to the respective average fidelities. We obtain fidelities of 99.15(3)% and 98.16(7)%, respectively, for the Parallel and Serial R_{ZX} gates. These fidelities were re-scaled by that of the identity operation, whose fidelity is 99.869(8)%, to discount the error in the Pauli twirling operations. As expected from the decoherence model introduced in the main text (Eq .(27)), we observe that $F_{\text{parallel}} \simeq F_{\text{serial}}^{0.514} = 99.05(3)\%$, with $t_S = 0.40$ μs, $t_P = 0.79$ μs, and $0.514 = t_P/t_S$ being the duration of the parallel gate relative to that of the serial gate. A similar result is obtained by using Eq. (27) of the main text, since F_0 is small. Moreover, we also observe that the fidelity uncertainty is noticeably smaller in the parallel version, reflecting more predictable behavior from the parallel gate.

N. T. Bronn, and G. T. Byrd, IEEE Transactions on Quantum Engineering , 1 (2022).

[6] A. Erhard, J. J. Wallman, L. Postler, M. Meth, R. Stricker, E. A. Martinez, P. Schindler, T. Monz, J. Emerson, and R. Blatt, Nature Communications **10**, 5347 (2019).

[7] J. J. Wallman and J. Emerson, Physical Review A **94**, 052325 (2016).

[8] J. A. Smolin, J. M. Gambetta, and G. Smith, Phys. Rev. Lett. **108**, 070502 (2012).

[9] D. Greenbaum, arXiv preprint arXiv:1509.02921 (2015).

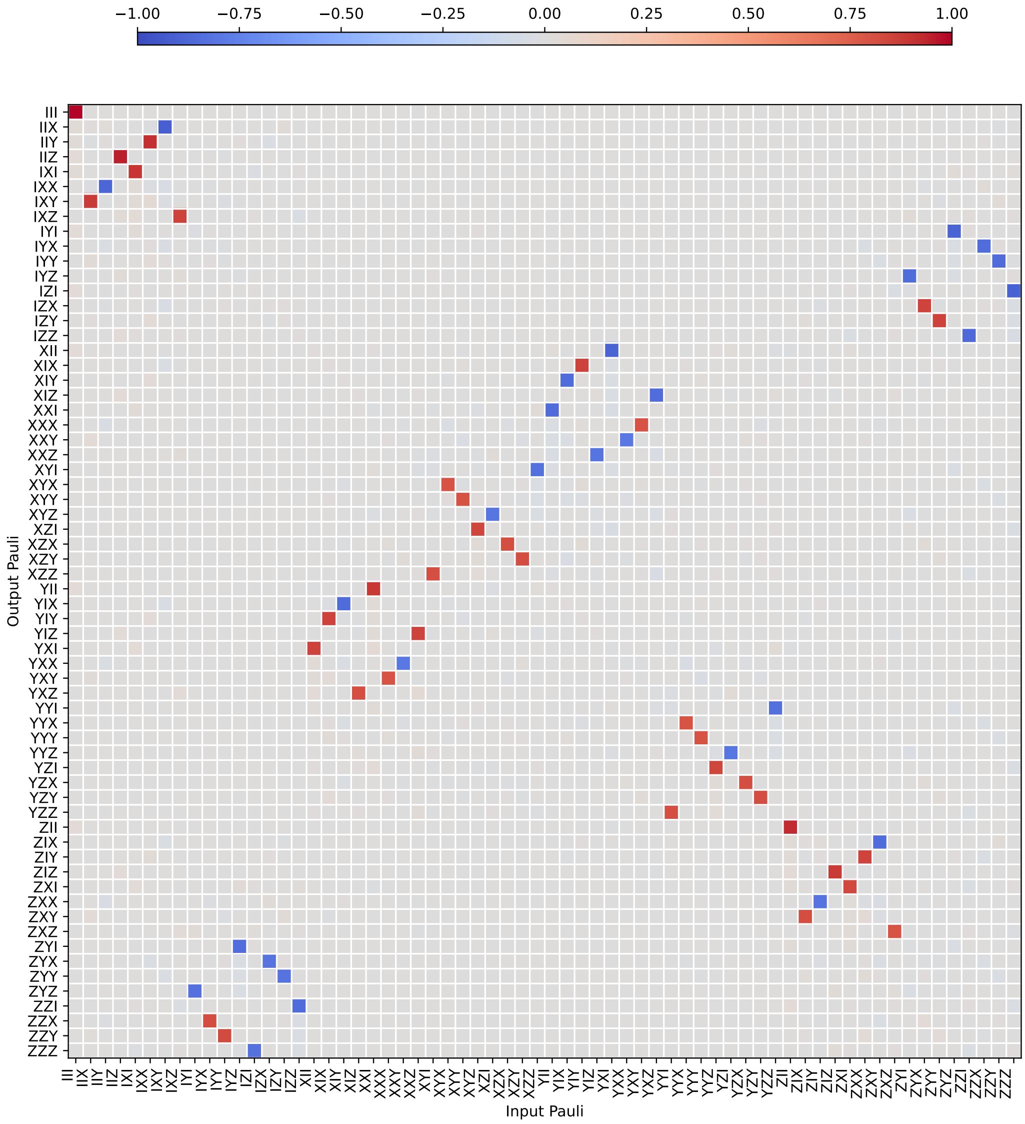

FIG. 4. Pauli Transfer Matrix of the Parallel $R_{ZX}(\pi/2)$, reconstructed from Process Tomography, as discussed in the main text.

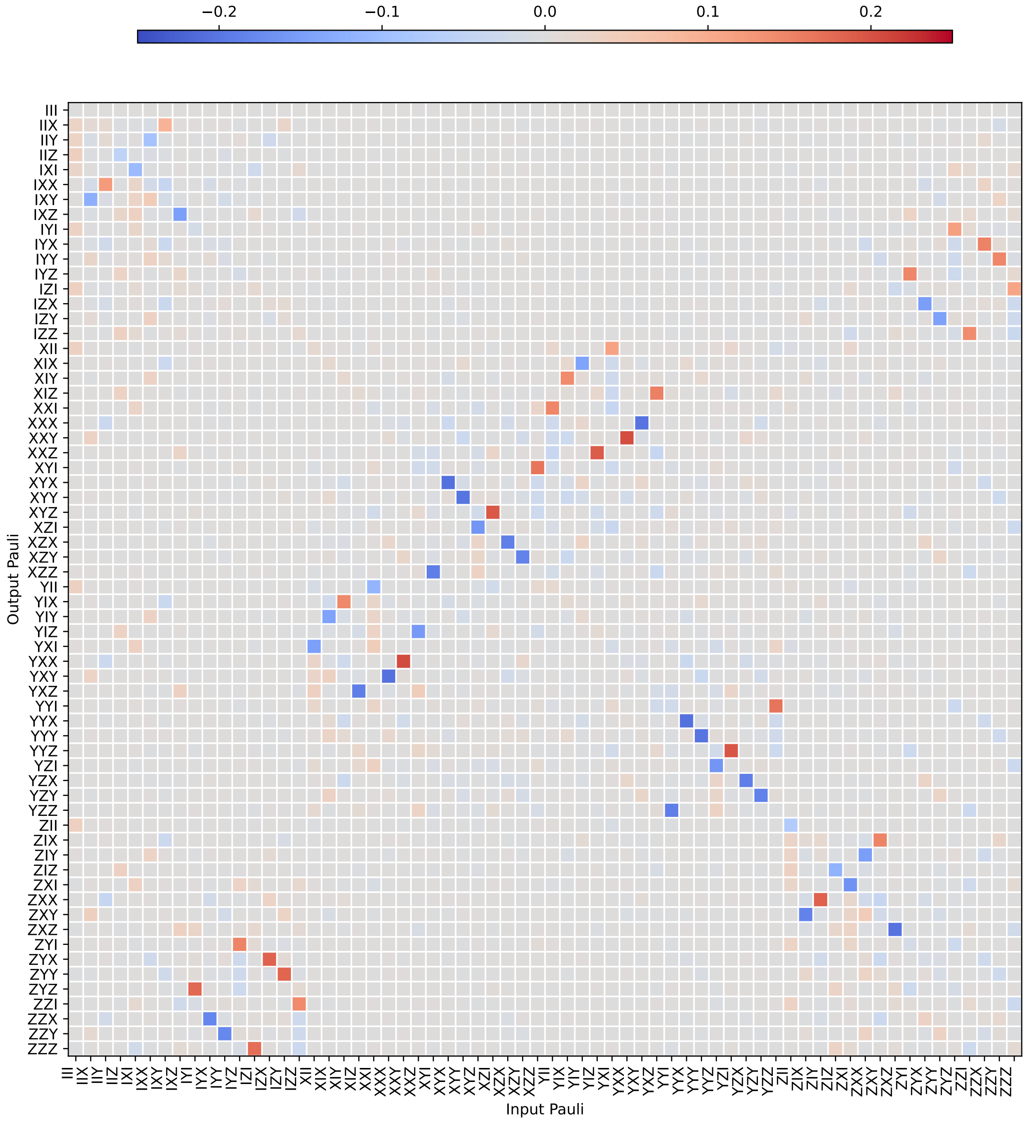

FIG. 5. Difference between the Pauli Transfer Matrix of the parallel version of of $R_{ZX}(\pi/2)$, reconstructed from Process Tomography, and its expected ideal PTM. We have changed the scaling for clarity. In this figure, we have not corrected for SPAM errors; therefore, since the PTM peak values are not exactly ± 1, this figure resembles the previous one, but with inverted colors.
